# Machine Learning to Predict In-Hospital Mortality in COVID-19 Patients Using Computed Tomography-Derived Pulmonary and Vascular Features

**DOI:** 10.3390/jpm11060501

**Published:** 2021-06-03

**Authors:** Simone Schiaffino, Marina Codari, Andrea Cozzi, Domenico Albano, Marco Alì, Roberto Arioli, Emanuele Avola, Claudio Bnà, Maurizio Cariati, Serena Carriero, Massimo Cressoni, Pietro S. C. Danna, Gianmarco Della Pepa, Giovanni Di Leo, Francesco Dolci, Zeno Falaschi, Nicola Flor, Riccardo A. Foà, Salvatore Gitto, Giovanni Leati, Veronica Magni, Alexis E. Malavazos, Giovanni Mauri, Carmelo Messina, Lorenzo Monfardini, Alessio Paschè, Filippo Pesapane, Luca M. Sconfienza, Francesco Secchi, Edoardo Segalini, Angelo Spinazzola, Valeria Tombini, Silvia Tresoldi, Angelo Vanzulli, Ilaria Vicentin, Domenico Zagaria, Dominik Fleischmann, Francesco Sardanelli

**Affiliations:** 1 Unit of Radiology, IRCCS Policlinico San Donato, Via Rodolfo Morandi 30, 20097 Milan, Italy; schiaffino.simone@gmail.com (S.S.); massimocressoni@gmail.com (M.C.); gianni.dileo77@gmail.com (G.D.L.); francesco.secchi@unimi.it (F.S.); francesco.sardanelli@unimi.it (F.S.); 2 Department of Radiology, School of Medicine, Stanford University, 300 Pasteur Drive, Stanford, CA 94305, USA; mcodari@stanford.edu (M.C.); d.fleischmann@stanford.edu (D.F.); 3 Department of Biomedical Sciences for Health, Università degli Studi di Milano, Via Luigi Mangiagalli 31, 20133 Milan, Italy; salvatore.gitto@unimi.it (S.G.); veronica.magni@unimi.it (V.M.); luca.sconfienza@unimi.it (L.M.S.); 4 IRCCS Istituto Ortopedico Galeazzi, Via Riccardo Galeazzi 4, 20161 Milan, Italy; albanodomenico@me.com (D.A.); carmelomessina.md@gmail.com (C.M.); 5 Department of Biomedicine, Neurosciences and Advanced Diagnostics, Section of Radiological Sciences, Università degli Studi di Palermo, Via del Vespro 127, 90127 Palermo, Italy; 6 Department of Diagnostic Imaging and Stereotactic Radiosurgery, C.D.I. Centro Diagnostico Italiano S.p.A., Via Simone Saint Bon 20, 20147 Milan, Italy; marco.ali@cdi.it; 7 Radiodiagnostics, Department of Diagnosis and Treatment Services, Azienda Ospedaliero Universitaria Maggiore della Carità, Corso Giuseppe Mazzini 18, 28100 Novara, Italy; robertoarioli91@gmail.com (R.A.); psc.dnn@gmail.com (P.S.C.D.); zenofalaschi@gmail.com (Z.F.); pascheale@gmail.com (A.P.); dzagaria19@gmail.com (D.Z.); 8 Postgraduate School in Radiodiagnostics, Università degli Studi di Milano, Via Festa del Perdono 7, 20122 Milan, Italy; emanuele.avola@unimi.it (E.A.); serena.carriero@unimi.it (S.C.); gianmarco.dellapepa@unimi.it (G.D.P.); 9 Unit of Interventional Radiology, Unit of Radiology, Fondazione Poliambulanza Istituto Ospedaliero, Via Leonida Bissolati 57, 25124 Brescia, Italy; claudio.bna@poliambulanza.it (C.B.); lore.monfa@gmail.com (L.M.); 10 Diagnostic and Interventional Radiology Service, ASST Santi Paolo e Carlo, Via Antonio di Rudinì 8, 20142 Milan, Italy; maurizio.cariati@asst-santipaolocarlo.it (M.C.); riccardo.foa@gmail.com (R.A.F.); silvia.3soldi@gmail.com (S.T.); 11 Emergency Department, ASST Crema—Ospedale Maggiore, Largo Ugo Dossena 2, 26013 Crema, Italy; fracrema01@gmail.com; 12 Unit of Radiology, Ospedale Universitario Luigi Sacco—ASST Fatebenefratelli Sacco, Via Giovanni Battista Grassi 74, 20157 Milan, Italy; flor.nicola@asst-fbf-sacco.it; 13 Unit of Interventional Radiology, Unit of Radiology, ASST Crema—Ospedale Maggiore, Largo Ugo Dossena 2, 26013 Crema, Italy; gioleati@gmail.com (G.L.); aspina@libero.it (A.S.); 14 High Speciality Center for Dietetics, Nutritional Education and Cardiometabolic Prevention, IRCCS Policlinico San Donato, Via Rodolfo Morandi 30, 20097 Milan, Italy; alexis.malavazos@gmail.com; 15 Department of Oncology and Hematology-Oncology, Università degli Studi di Milano, Via Festa del Perdono 7, 20122 Milan, Italy; giovanni.mauri@unimi.it (G.M.); angelo.vanzulli@unimi.it (A.V.); 16 Division of Interventional Radiology, IEO—Istituto Europeo di Oncologia IRCCS, Via Giuseppe Ripamonti 435, 20141 Milan, Italy; 17 Division of Breast Radiology, IEO—Istituto Europeo di Oncologia IRCCS, Via Giuseppe Ripamonti 435, 20141 Milan, Italy; filippopesapane@gmail.com; 18 Department of General and Emergency Surgery, ASST Crema—Ospedale Maggiore, Largo Ugo Dossena 2, 26013 Crema, Italy; edo.87@hotmail.it; 19 ASST Grande Ospedale Metropolitano Niguarda, Piazza dell’Ospedale Maggiore 3, 20162 Milan, Italy; valeria.tombini@ospedaleniguarda.it (V.T.); ilaria.vicentin@unimi.it (I.V.); 20 Cardiovascular Institute, 265 Campus Drive, Stanford University, Stanford, CA 94305, USA

**Keywords:** COVID-19, lung, pulmonary artery, tomography, X-ray computed, machine learning, support vector machine, neural networks, computer, prognosis

## Abstract

Pulmonary parenchymal and vascular damage are frequently reported in COVID-19 patients and can be assessed with unenhanced chest computed tomography (CT), widely used as a triaging exam. Integrating clinical data, chest CT features, and CT-derived vascular metrics, we aimed to build a predictive model of in-hospital mortality using univariate analysis (Mann–Whitney *U* test) and machine learning models (support vectors machines (SVM) and multilayer perceptrons (MLP)). Patients with RT-PCR-confirmed SARS-CoV-2 infection and unenhanced chest CT performed on emergency department admission were included after retrieving their outcome (discharge or death), with an 85/15% training/test dataset split. Out of 897 patients, the 229 (26%) patients who died during hospitalization had higher median pulmonary artery diameter (29.0 mm) than patients who survived (27.0 mm, *p* < 0.001) and higher median ascending aortic diameter (36.6 mm versus 34.0 mm, *p* < 0.001). SVM and MLP best models considered the same ten input features, yielding a 0.747 (precision 0.522, recall 0.800) and 0.844 (precision 0.680, recall 0.567) area under the curve, respectively. In this model integrating clinical and radiological data, pulmonary artery diameter was the third most important predictor after age and parenchymal involvement extent, contributing to reliable in-hospital mortality prediction, highlighting the value of vascular metrics in improving patient stratification.

## 1. Introduction

Since the inception of the severe acute respiratory syndrome coronavirus 2 (SARS-CoV-2) pandemic, chest computed tomography (CT) has been widely used both as a triaging test on emergency department admission [1,2,3]—especially in the case of unavailability of immediate result of reverse transcriptase-polymerase chain reaction (RT-PCR)—and as a diagnostic tool to monitor Coronavirus Disease 2019 (COVID-19) pneumonia [1,2].

The widely recognized systemic prothrombotic profile of COVID-19, swiftly linked with worse outcomes [4,5,6,7,8,9,10,11], directly affects pulmonary vasculature [11,12,13,14,15,16,17,18,19,20]. In hospitalized COVID-19 patients with moderate and severe disease, CT-detected lung parenchymal involvement [21,22] has been associated with varying degrees of pulmonary arterial vascular damage [10,11,12,13,14,15], which disrupts vascular dynamics and increases pulmonary artery (PA) pressure [23,24,25,26,27,28,29]. Of note, bedside ultrasound examinations performed during hospitalization have hinted the prognostic value of indirect signs of impaired pulmonary circulation, such as right ventricular longitudinal strain and increased end-diastolic chamber size [29,30]. However, these parameters are encumbered by operator dependency and potential overlap with other pre-existing causes of right ventricular dysfunction [31]; moreover, the routine performance of echocardiography in the triage of COVID-19 patients during pandemic peaks could prove challenging, due to the high volume of patients referred to emergency departments.

Rather than identifying thrombotic phenomena as a byproduct of pulmonary embolism, ever-increasing evidence points to a direct origin of thrombosis in arterial lung vasculature [14,32,33], highlighting the need for a more accurate characterization of these phenomena in their true location [34].

Diagnosis of pulmonary arterial thrombosis requires contrast-enhanced CT angiography, which implies the intravenous administration of iodinated contrast agents [35]. However, alterations of pulmonary vascular metrics also detectable with unenhanced CT, such as the enlargement of the PA and an increased ratio between diameters of the PA and of the ascending aorta (AA), are known indirect signs of pulmonary hypertension caused by fibrotic or thromboembolic processes [36,37,38,39,40,41,42]. Notably, these pulmonary vascular metrics have been demonstrated to be altered also in COVID-19 patients—when compared to previous values measured on CT scans acquired before the SARS-CoV-2 pandemic—and to carry prognostic implications [25,26,27]. Since unenhanced chest CT is still widely performed in COVID-19 patients, the integration of CT-derived vascular features with other readily available clinical and imaging parameters could improve the stratification of COVID-19 patients on emergency department admission, potentially improving patient management and prognosis.

This multicenter study, conducted in six different hospitals in northern Italy during the first pandemic peak of 2020, aims to evaluate the prognostic power of a machine learning model integrating imaging features of lung parenchyma and vasculature with clinical data of COVID-19 patients routinely retrieved on emergency department admission.

## 2. Materials and Methods

### 2.1. Study Population

This multicenter observational study was conducted in six different institutions in northern Italy: Azienda Ospedaliero-Universitaria Maggiore della Carità, Novara (Center 1); ASST Grande Ospedale Metropolitano Niguarda, Milano (Center 2); Fondazione Poliambulanza Istituto Ospedaliero, Brescia (Center 3); ASST Crema—Ospedale Maggiore, Crema (Center 4); ASST Santi Paolo e Carlo, Milano (Center 5); and IRCCS Istituto Ortopedico Galeazzi, Milano (Center 6). Approval for this retrospective study was obtained from the Ethics Committee of the coordinating center IRCCS Policlinico San Donato (Comitato Etico IRCCS Ospedale San Raffaele, protocol code COVID19-TCretro, protocol number 77/INT/2020, approved 5 May 2020). Study-specific informed consent was waived due to the retrospective nature of the study.

We included patients hospitalized in each of these institutions during the study period (21 February to 30 April 2020) with RT-PCR-confirmed SARS-CoV-2 infection and unenhanced chest CT performed on emergency department admission. Authors from each center reviewed their own institutional electronic databases to retrieve patients’ clinical data, including: demographics, symptoms, partial pressure of oxygen in arterial blood (PaO2), comorbidities, smoking history, body mass index, and white blood cells, lymphocytes, platelets, and lactate dehydrogenase values. For outcome assessment, censoring was applied on 1 June 2020, when all patients had either been discharged or had died during hospitalization.

### 2.2. Image Acquisition and Analysis

Unenhanced chest CT scans were performed in all six centers in supine position, during a single inspiratory breath-hold condition when possible. Table 1 shows the technical characteristics of the scanners and acquisition parameters.

**Figure 1:**
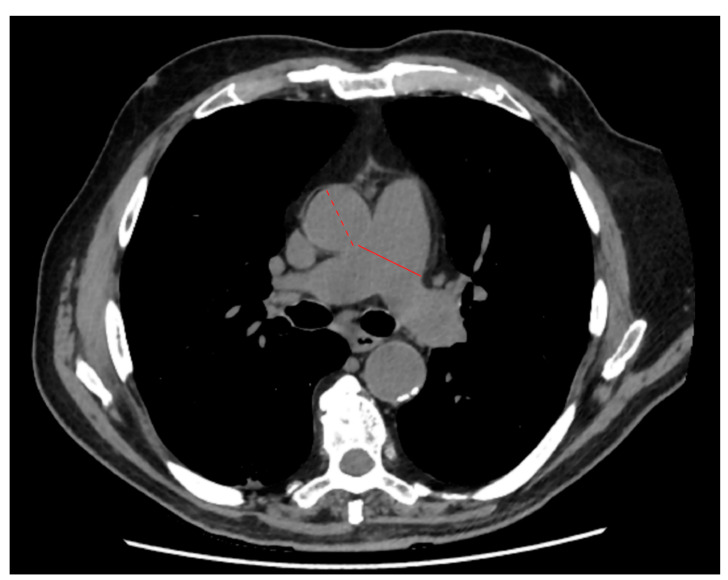
Example of axial slice selected at the level of pulmonary arterial main trunk bifurcation. The solid and dashed lines identify the maximum diameters of the pulmonary artery and ascending aorta, respectively.

Radiologists with 7 to 32 years of experience blindly reviewed CT images from their own institution, assessing lung parenchyma and the presence of pleural effusion and mediastinum lymph nodes with short-axis diameters larger than 1 cm [43]. Lung parenchymal involvement was qualitatively assessed by radiologists on their own institutional digital imaging and communications in medicine (DICOM) viewer, considering the presence of parenchymal involvement signs, (i.e., ground-glass opacities (GGOs), consolidations, and crazy paving pattern) and the volumetric extent of parenchymal involvement, assessed according to Chung et al. [21]: 0% (absent, 0); 1–25% (minimal, 1); 26–50% (mild, 2); 51–75% (moderate, 3); and over 75% (severe, 4). As described by Wells et al. [44], PA and AA maximum diameters were measured on a single axial slice selected at the level of pulmonary arterial main trunk bifurcation (Figure 1).

### 2.3. Data Preprocessing and Initial Feature Selection

To build a predictive model of in-hospital mortality, we selected among available features those with no more than 25% missing data. In these selected features, residual missing data were imputed with the mean value of the respective feature. Data were randomly split into a training/validation set (85%) and a testing set (15%). In each set, all features were rescaled to have null mean and unitary variance.

To prevent overfitting, we used training/validation data to perform a two-step feature selection process. First, we removed highly correlated features by thresholding the feature correlation matrix, setting the absolute Pearson’s correlation threshold value at 0.7. Then, we used the least absolute shrinkage and selection operator (LASSO) to compute feature importance, i.e., the absolute value of the LASSO regression coefficients [45,46]. The best α value was determined using a ten-fold cross-validation process. Selected features were used to develop several machine learning models using both support vector machines (SVM) and multilayer perceptrons (MLP).

### 2.4. Support Vector Machines

We initialized the SVM classifier with linear kernel, C value = 1, and balanced class-weights using all selected features. Then, we performed a systematic hyperparameter grid-search to find: the optimal SVM kernel among linear, radial basis function, polynomial, and sigmoid kernels; the best C values among 100 values linearly sampled in the range 1–8; and the best γ value (for nonlinear kernels) among ten logarithmically-sampled (base 10) values in the range 10^−10^–10^−1^. Each candidate model (*n* = 310) was validated using a ten-fold cross-validation process, accounting for 31,000 fits. The best model was defined as the one that maximized the average F1 score in validation data. F1 score was selected as a scoring metric to consider class imbalance. Finally, the best model was refitted on the entire training/validation set.

Given this initial model and its optimal C (C_opt_) and γ (γ_opt_) hyperparameters, we performed an importance-based backward feature selection. We optimized SVM hyperparameters for each feature subset using a second systematic grid search strategy, further fine-tuning C and γ values in narrower ranges. The new search-grid was defined by selecting 25 C values linearly sampled in the range (C_opt_ ± 2), while 50 γ values were logarithmically sampled in the range $(10^{\log\left(\gamma_{\mathrm{opt}}-1\right)}$, $10^{\log\left(\gamma_{\mathrm{opt}}+1\right)}$) Each one of the 1250 candidate models was validated using a ten-fold cross-validation process. Again, the best model (i.e., the one that maximized the average F1 score in validation data) was finally refitted over the entire training/validation set.

### 2.5. Multilayer Perceptrons

After selecting all features with a non-zero LASSO coefficient, we further selected features by keeping the most important ones that allowed us to meet the rule of thumb of at least ten events in our training/validation set for each feature included in our model [47,48].

The general MLP architecture is summarized in Table 2. We initialized the hidden unit numbers N and M values to 7 and 5, respectively. To prevent dying rectified linear unit (ReLU) issues, we used LeakyReLU [49] as activation function (α = 0.03), while weights were initialized using the method proposed by He et al. [50]. To improve model generalizability and prevent overfitting, we added a dropout layer after each fully connected layer with a dropout rate equal to 0.2. The last layer of the perceptron comprised a single unit with a sigmoid activation function to encode patient mortality. The initial learning rate was set to 0.01, being then decreased during training using an exponential decay schedule of 1000 steps and a base of 0.5. We set binary cross-entropy as the loss function, adopted the Adam optimizer [51], and set a default batch-size value of 32. The F1 score, the area under the curve (AUC) at receiving operator characteristic (ROC) analysis, accuracy, precision, and recall were selected as the performance metrics. The training stopped if the loss function did not decrease for 25 epochs, then the best weights corresponding to the loss function minimum were restored. The maximum number of epochs was set to 1000.

Given this model and its hyperparameters, we performed a systematic grid-search ten-fold cross-validation to optimize the following hyperparameters: the batch size in (4, 16, 32, 64); the dropout rate in (0.1, 0.2, 0.4); the starting learning rate in (0.1, 0.01, 0.001); the number of hidden units in the second and third layers (N) in (5, 7, 10); and the number of hidden units in the fourth and fifth layers (M) in (3, 5, 7). Each one of the 324 candidate MLPs was validated using a ten-fold cross-validation process (3240 fits). The best model (i.e., the one that maximized the average F1 score in validation data) was finally refitted over the entire training/validation set.

### 2.6. Statistical Analysis

Due to their nonparametric distribution, assessed with the Shapiro–Wilk test, continuous variables were reported as median with interquartile range (IQR), while categorical variables as total number and percentage. The Mann–Whitney *U* test was used to compare means from different groups for continuous variables during univariate explorative analysis. Statistical analyses were performed using SPSS v.26.0 (IBM SPSS Inc., Chicago, IL, USA), and *p* values < 0.05 were considered statistically significant. All SVMs and MLPs were developed using python v.3.7.7 [52], sklearn v.0.23.1 [53], and tensorflow-gpu v.2.2.0 [54] on a laptop with an Intel Core i7-6500 CPU (2.50 GHz), 8 GB of RAM, and a Nvidia GeForce 940MX GPU.

## 3. Results

### 3.1. Population Characteristics

The study population included 897 COVID-19 patients (608 males, 68%; median age 66 years, IQR 55–77 years), hospitalized in six different centers from 21 February to 30 April 2020. A total of 270 patients came from Center 1, 197 from Center 2, 194 from Center 3, 144 from Center 4, 80 from Center 5, and 12 from Center 6.

Symptoms, comorbidities, height, weight, and laboratory parameters were assessed on emergency department admission and are reported in Table 3, alongside CT-derived lung and vascular features. The main symptoms observed on emergency department admission were fever (500/897 patients, 56%), cough (277/897 patients, 31%), and dyspnea (229/897 patients, 26%). During hospitalization, which lasted a median of 7 days (IQR 4–13 days), 133/897 patients (15%) were admitted to intensive care units (ICU). Low molecular weight heparin (LMWH) was administered to 296/897 patients (33%): to 88/296 (30%) at therapeutic dosage, to 177/296 (60%) at prophylactic dosage, while for the remaining 31/296 (10%) dosage data was not available. At censoring for outcome assessment (June 1, 2020), we found that 229/897 (26%) patients had died during hospitalization, while the remaining 668/897 (74%) had been discharged.

### 3.2. Explorative Univariate Analysis of Pulmonary Vascular Features

The overall median PA maximum diameter was 28 mm (25–30 mm) and median AA 34 mm (32–37 mm). Patients who died during hospitalization showed significantly higher median PA maximum diameter (29.0 mm, IQR 26.0–32.0 mm) compared to patients who survived (27.0 mm, IQR 25.0–30.0 mm, *p* < 0.001), and significantly higher median AA maximum diameter (36.6 mm, IQR 34.0–39.0 mm, versus 34.0 mm, IQR 31.0–36.0 mm, *p* < 0.001).

### 3.3. Support Vector Machines and Multilayer Perceptrons

**Figure 2:**
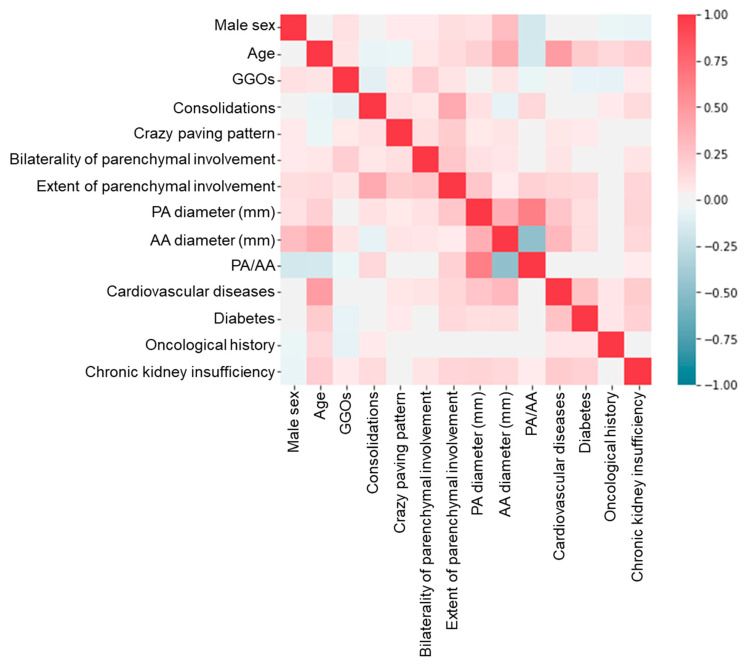
Feature correlation matrix, the color gradient representing the Pearson’s correlation coefficient value. GGOs: ground glass opacities; PA: pulmonary artery; AA: ascending aorta.

**Figure 3:**
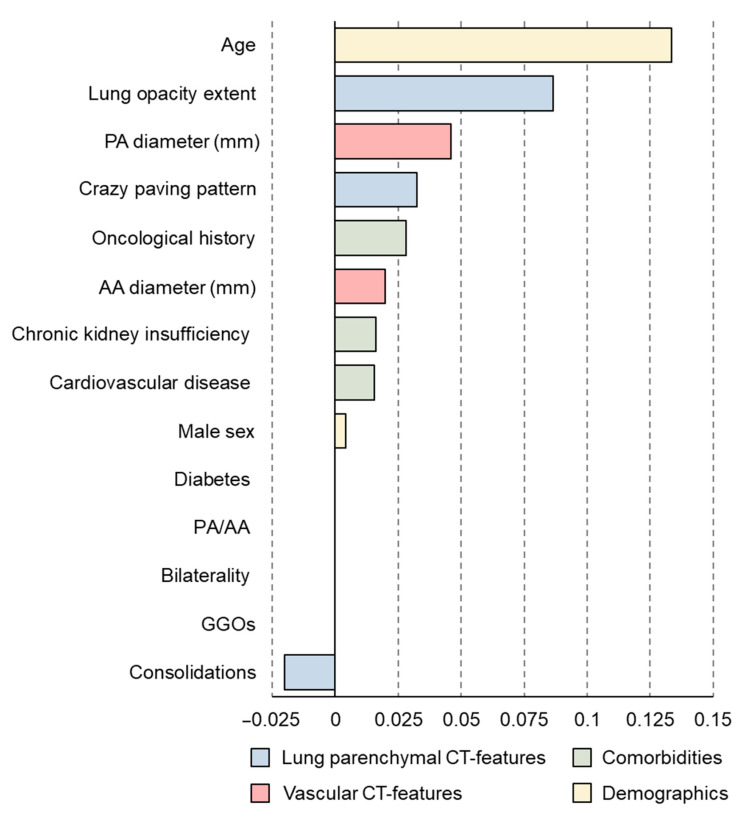
Coefficient of the least absolute shrinkage and selection operator (LASSO) regression in training/validation data. PA: pulmonary artery; AA: ascending aorta; GGOs: ground glass opacities.

After discarding features with a percentage of missing data higher than 25%, 14 features were selected for further processing (Table 3). After this selection step, residual missing data were clustered in 223 patients, who had almost only input data and were therefore also removed. Only 674 out of the initial 897 patients (75%) were therefore used to develop all machine learning models. The percentage of missing data ranged from 0% to 5% in the remaining features (median 0.4%, IQR 0.1–3.2%). After the initial random database split, our training/validation dataset was composed of 572 patients, 160 of whom (28%) died during hospitalization, while the testing set accounted for 102 patients, 30 of whom died during hospitalization (29%). No features were excluded after Pearson’s correlation matrix thresholding (Figure 2). Obtained LASSO coefficients for all initial features are reported in Figure 3.

**Figure 4:**
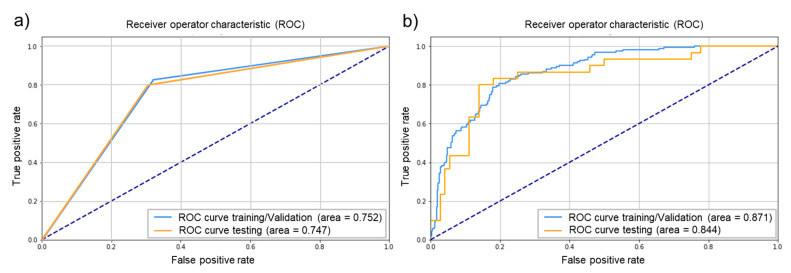
Receiver operator characteristic curves of the best support vector machine (**a**) and multilayer perceptron (**b**) models.

Fourteen SVM models were developed using different feature subsets. The best SVM model took 10 features as inputs (Table S1). The best model had a radial basis function kernel, with C equal to 5.924 and γ equal to 6.06 × 10^−5^. After the model was refitted on the entire training/validation dataset, the final model performances on testing data were: an F1 score of 0.632, an AUC of 0.747, a precision of 0.522, and a recall of 0.800. All SVMs had radial basis function kernels, their other hyperparameters and performance being reported in Table S1, while the ROC curve of the best SVM model is depicted in panel a of Figure 4.

Due to the high number of events in the training/validation set, MLPs were built using all features with a non-null LASSO coefficient (*n* = 10). Therefore, all developed MLPs have the same input features as the best SVM model. Among the 324 models resulting from the systematic hyperparameter grid search, the best MLP had the following hyperparameters: N and M equal to 7 and 3 respectively; a batch-size of 4; a dropout rate equal to 0.1; and a starting learning rate equal to 0.01. After refitting the best model on the entire training/validation dataset, the final performance on testing data were: an F1 score of 0.618, an AUC of 0.844, a precision of 0.680, and a recall of 0.567. The ROC curve of the best MLP is depicted in panel b of Figure 4.

## 4. Discussion

In this retrospective multicenter study, we developed a predictive model of in-hospital mortality for COVID-19 patients, using clinical and radiological data acquired on emergency department admission. The CT data of 674 patients from six hospitals in northern Italy were used to extract pulmonary parenchymal and vascular features. While machine learning models centered on clinical and imaging data have been proposed to aid both COVID-19 diagnosis and severity stratification [55,56,57,58,59], the inclusion of vascular features stems from an ever-larger corpus of observations that link vascular (particularly endothelial) impairment in COVID-19 [4,5,6,7,8,9] to pulmonary thromboembolism [11,12,13,16,17,18,19,20,23] and gross damage to pulmonary arterial vessels [25,26,27].

In a preliminary study on a limited sample size [26] we already observed that COVID-19 patients have a PA maximum diameter greater than that measured in previous CTs performed for non-cardiovascular reasons. In that study, enlarged PA diameter was also associated to death during hospitalization [26]. These observations were confirmed by the present study already through the explorative univariate analysis of pulmonary vascular features, where we found that patients who died during hospitalization had a significantly higher PA maximum diameter. Further, as shown in Figure 3, the LASSO regression coefficient for the PA maximum diameter was the third highest among investigated predictors of in-hospital death, after age and the extent of lung parenchymal involvement. It is worth noting that lung consolidation, appearing in the later stages of COVID-19 pneumonia, seems to be associated with better prognosis, as highlighted by the negative LASSO regression coefficient. This apparently counterintuitive result, already hinted at by a few studies [60,61], can be explained by considering that extensive parenchymal involvement in COVID-19 patients most frequently leads to in-hospital death, leaving no time for pulmonary damage progression to consolidation. Indeed, consolidation was present in almost 50% of patients from our cohort but was sparsely distributed and was associated with GGOs in 91% of cases, pointing out how hospital admission occurred at relatively early stages of COVID-19 pneumonia.

The prognostic role of the PA maximum diameter also shows how the early microvascular damage caused by SARS-CoV-2 infection may represent one of the most insidious aspects of severe COVID-19 presentations [10]. Notably, SARS-CoV-2-induced microthrombosis in distal branches of the PA can start the disruption of the already fragile coagulation profile of these patients [5,8,9], but can be difficult to detect even on contrast-enhanced CT scans [23]. Conversely, the PA diameter is an indirect metric of the thromboembolic profile of COVID-19 patients that can be easily obtained from unenhanced CT scans. Its integration into a reliable prognostic model further highlights how features extracted from CT scans performed without the administration of a contrast agent carry a substantial prognostic potential in COVID-19 patients, as already hinted at by other studies [62,63].

This study was performed at the early stage of the SARS-CoV-2 pandemic, when prophylactic LMWH was not yet routinely administered. This represents the main limitation of our study, preventing the investigation of the impact of appropriate treatment—targeting vascular damage—on COVID-19 patients’ prognosis. Moreover, shortages in ICU beds affecting all hospitals in northern Italy during the first pandemic peak [64] frequently restricted ICU admission to relatively younger patients without comorbidities, preventing us from assessing ICU admission predictive models, since these would have been biased by such patient selection. Of note, this logistical stress on healthcare systems also precluded the inclusion of young patients (which were prevalently treated at home), hindered data collection on admission, and likely impacted the prognosis of the included patients: all these aspects must be carefully considered when evaluating the generalizability of our results. From a technical point of view, the main limitation of our study is the lack of a longitudinal and independent test set, as we randomly sampled test patients from the original multicenter cohort. Since we aimed first to maximize the heterogeneity of the training set as much as possible, using retrospective data collected from all centers, a robust generalizability assessment will need to be conducted prospectively on an independent test set. Although commonly reported in published literature [25,37,38,39,44], another technical limitation is represented by the manual measurement of the diameters of great vessels on axial scans: a better approach could be represented by acquiring vessel area measurements on reformatted oblique reconstructions considering the vessel axis [42]. Moreover, we focused on a single feature selection technique (i.e., the LASSO) and on few machine learning models (i.e., SVMs and MLPs) to develop the in-hospital mortality predictor, with a comparably small batch size in MLPs, although supported by results of systematic hyperparameter search. Finally, the use of the mean feature value as an imputation technique may also lead to suboptimal performance. Indeed, the use of multiple imputations or machine learning based imputation techniques could improve the predictive power of developed classifiers.

In conclusion, our study shows how a predictive model integrating simple clinical and radiological data acquired on admission, such as the PA/AA ratio on unenhanced CT scans, allowed to predict in-hospital death in COVID-19 patients, highlighting the major role of pulmonary vascular involvement in the stratification of COVID-19 patients.

## Figures and Tables

**Table 1:** Center-specific technical characteristics of the CT scanners and acquisition parameters.

Center	Location	Vendor	Model	Acquired Slices	Slice Thickness (mm)	Tube Voltage (kVp)
Azienda Ospedaliero-Universitaria Maggiore della Carità	Novara	Philips Healthcare	Ingenuity Core	128	1	120
ASST Grande Ospedale Metropolitano Niguarda	Milano	Siemens Healthineers	Somatom Definition Edge	128	1.5	120
Fondazione Poliambulanza Istituto Ospedaliero	Brescia	General Electric Healthcare	LightSpeed RT 16	16	1.25	120
ASST Crema—Ospedale Maggiore	Crema	Canon	Aquilion CXL	64	1.5	135
		General Electric Healthcare	Revolution EVO	64	1.25	120
ASST Santi Paolo e Carlo	Milano	General Electric Healthcare	LightSpeed RT 16	16	1.25	120
IRCCS Istituto Ortopedico Galeazzi	Milano	Siemens Healthineers	Somatom Definition AS 64	64	1.5	120

**Table 2:** Multilayer perceptron summary architecture.

Layer	Number of Hidden Units	Trainable Parameters
Dense_1	Number of selected features (f)	f × (f + 1)
Dropout_1	Number of selected features (f)	0
Dense_2	N	N × (f + 1)
Dropout_2	N	0
Dense_3	N	N × (N + 1)
Dropout_3	N	0
Dense_4	M	M × (N + 1)
Dropout_4	M	0
Dense_5	M	M × (M + 1)
Dropout_5	M	0
Dense_6	1	M + 1

N: number of hidden units in the second and third layer of the multilayer perceptron; M: number of hidden units in the fourth and fifth layer of the multilayer perceptron.

**Table 3:** Demographic, clinical, and imaging characteristics of the two sets of patients.

Variable		Variable Type	Overall (897 Patients)	Training/Validation Set (572 Patients)	Test Set (102 Patients)
**Demographics**					
Sex		Categorial	608 M/289 F	389 M/183 F	66 M/36 F
Age (years)		Continuous	66.2 (55.1–76.5)	66.9 (55.9–77.0)	66.7 (52.7–79.2)
**Comorbidities**					
Cardiovascular diseases		Dichotomic	433 (48%)	320 (56%)	53 (52%)
Diabetes		Dichotomic	151 (17%)	113 (20%)	19 (19%)
Oncological history		Dichotomic	76 (8%)	52 (9%)	10 (10%)
Chronic kidney insufficiency		Dichotomic	52 (6%)	45 (8%)	3 (3%)
**Outcome**					
Deceased patients		Dichotomic	229 (26%)	160 (28%)	30 (29%)
**CT findings and features**					
**Lung parenchyma**	Ground-glass opacities	Dichotomic	681 (76%)	504 (93%)	90 (92%)
	Consolidations	Dichotomic	434 (48%)	271 (50%)	53 (54%)
	Crazy paving pattern	Dichotomic	194 (22%)	162 (30%)	36 (37%)
	Extent of parenchymal involvement *	Discrete	2 (1–3)	2 (1–3)	2 (1–3)
	Bilateral parenchymal involvement	Dichotomic	631 (70%)	502 (93%)	86 (88%)
**Vascular features**	PA diameter (mm)	Continuous	28.0 (25.0–30.0)	28.0 (25.0–30.1)	28.0 (25.0–30.3)
	AA diameter (mm)	Continuous	34.0 (32.0–37.0)	35.0 (32.0–37.0)	34.0 (31.0–37.4)
	PA/AA ratio	Continuous	0.81 (0.73–0.89)	0.81 (0.73–0.90)	0.81 (0.74–0.89)

All descriptive statistics are reported as median and interquartile ranges or frequencies for continuous and dichotomic/discrete variables, respectively. M: males; F: females; PA: pulmonary artery; AA: ascending aorta. * Semi-quantitatively assessed from 0 to 4, according to Chung et al. [21], as follows: 0% = 0 (absent); 1–25% = 1 (minimal); 26–50% = 2 (mild); 51–75% = 3 (moderate); and over 75% = 4 (severe).

## Data Availability

We will be able to make available deidentified individual participant data that underlie the results reported in this article, beginning 12 months and ending 24 months following article publication. We will consider requests from researchers who will provide a methodologically sound proposal and will share data needed to achieve the aims declared in the approved proposal. Proposals may be submitted up to 36 months following article publication. After 24 months the data will be available in the data warehouse of the coordinating center but without investigator support other than deposited metadata. Information regarding submitting proposals and accessing data may be obtained by email inquiry to the first/corresponding author at andrea.cozzi1@unimi.it.

## Supplementary Materials

The following are available online at https://www.mdpi.com/article/10.3390/jpm11060501/s1, Table S1: Input features, hyperparameters, and performance of the support vector machines developed as a result of the LASSO-importance-based backward feature selection.
