# Supplementary material for: Machine Learning to Predict In-Hospital Mortality in COVID-19 Patients Using Computed Tomography-Derived Pulmonary and Vascular Features
Source: J Pers Med. 2021 Jun 3;11(6):501. doi: 10.3390/jpm11060501 (PMC8230339; doi:10.3390/jpm11060501)
Supplement: Supplementary file 1 [file jpm-11-00501-s001.zip › jpm-1219874-SI.pdf]

Journal of Personalized Medicine

MDPI

Table S1. Input features, hyperparameters and performance of the support vector machines developed as a result of the LASSO-importance-based backward feature selection.

		Hyperparameters		Training (10-fold CV)			Validation (10-fold CV)			Training/validation			Testing		
N_{if}	Selected features	Final C	Final γ	AUC	Pr	Rc	AUC	Pr	Rc	AUC	Pr	Rc	AUC	Pr	Rc
14	Male sex, age, GGOs, consolidations, crazy paving, bilaterality, lung opacity extent, PA diameter, AA diameter, PA/AA, cardiovascular disease, diabetes, oncological history, chronic kidney insufficiency	7.258	5.52×10^{-5}	0.814 ± 0.006	0.492 ± 0.013	0.774 ± 0.025	0.802 ± 0.052	0.506 ± 0.080	0.769 ± 0.115	0.726	0.494	0.750	0.754	0.533	0.800
10	Male sex, age, consolidations, crazy paving, lung opacity extent, PA diameter, AA diameter, cardiovascular disease, oncological history, chronic kidney insufficiency	5.924	6.06×10^{-5}	0.817 ± 0.006	0.499 ± 0.014	0.806 ± 0.022	0.806 ± 0.047	0.509 ± 0.076	0.794 ± 0.101	0.752	0.500	0.825	0.747	0.522	0.800
9	Age, consolidations, crazy paving, lung opacity extent, PA diameter, AA diameter, cardiovascular disease, oncological history, chronic kidney insufficiency	4.258	9.70×10^{-5}	0.817 ± 0.005	0.499 ± 0.014	0.793 ± 0.028	0.806 ± 0.047	0.511 ± 0.065	0.800 ± 0.096	0.746	0.487	0.831	0.747	0.522	0.800
8	Age, consolidations, crazy paving, lung opacity extent, PA diameter, AA diameter, oncological history, chronic kidney insufficiency	6.258	5.52×10^{-5}	0.822 ± 0.006	0.479 ± 0.023	0.765 ± 0.053	0.812 ± 0.050	0.499 ± 0.085	0.775 ± 0.109	0.717	0.492	0.725	0.789	0.600	0.800
7	Age, consolidations, crazy paving, lung opacity extent, PA diameter, AA diameter, oncological history	5.758	2.26×10^{-4}	0.829 ± 0.007	0.492 ± 0.012	0.746 ± 0.014	0.819 ± 0.054	0.499 ± 0.092	0.738 ± 0.096	0.728	0.498	0.750	0.792	0.581	0.833
6	Age, consolidations, crazy paving, lung opacity extent, PA diameter, oncological history	8.091	2.48×10^{-4}	0.832 ± 0.007	0.505 ± 0.012	0.775 ± 0.025	0.821 ± 0.057	0.500 ± 0.073	0.763 ± 0.092	0.737	0.500	0.775	0.772	0.590	0.767

5	Age, crazy paving, lung opacity extent, PA diameter, oncological history	6.758	7.32 ×10^{-5}	0.820 ± 0.006	0.520 ± 0.023	0.701 ± 0.041	0.809 ± 0.055	0.533 ± 0.084	0.713 ± 0.085	0.732	0.509	0.744	0.746	0.583	0.700
4	Age, crazy paving, lung opacity extent, PA diameter	4.758	1.55×10^{-4}	0.819 ± 0.006	0.502 ± 0.013	0.742 ± 0.022	0.811 ± 0.055	0.517 ± 0.064	0.750 ± 0.112	0.724	0.494	0.744	0.765	0.575	0.767
3	Age, lung opacity extent, PA diameter	4.591	1.17 ×10^{-4}	0.814 ± 0.006	0.464 ± 0.011	0.800 ± 0.035	0.810 ± 0.058	0.475 ± 0.070	0.800 ± 0.073	0.713	0.467	0.763	0.754	0.533	0.800
2	Age, lung opacity extent	7.758	2.48×10^{-4}	0.812 ± 0.006	0.469 ± 0.013	0.838 ± 0.010	0.808 ± 0.052	0.469 ± 0.060	0.838 ± 0.089	0.737	0.475	0.831	0.774	0.531	0.867
1	Age	7.258	2.48×10^{-4}	0.774 ± 0.005	0.426 ± 0.004	0.862 ± 0.013	0.773 ± 0.049	0.429 ± 0.035	0.863 ± 0.073	0.702	0.427	0.844	0.725	0.464	0.867

All models used a radial basis function kernel. Hyperparameters were optimized using a systematic grid search combined with a 10-fold cross-validation. CV: cross-validation; AUC: area under the curve; N_{if}: number of input features; Pr: precision; Rc: recall.
